# Protein sequence classification using feature hashing

**DOI:** 10.1186/1477-5956-10-S1-S14

**Published:** 2012-06-21

**Authors:** Cornelia Caragea, Adrian Silvescu, Prasenjit Mitra

**Affiliations:** 1 Information Sciences and Technology, Pennsylvania State University, University Park, PA, USA; 2 Naviance Inc., Oakland, CA, USA

## Abstract

Recent advances in next-generation sequencing technologies have resulted in an exponential increase in the rate at which protein sequence data are being acquired. The *k*-gram feature representation, commonly used for protein sequence classification, usually results in *prohibitively *high dimensional input spaces, for large values of *k*. Applying data mining algorithms to these input spaces may be intractable due to the large number of dimensions. Hence, using dimensionality reduction techniques can be crucial for the performance and the complexity of the learning algorithms. In this paper, we study the applicability of feature hashing to protein sequence classification, where the original high-dimensional space is "reduced" by hashing the features into a low-dimensional space, using a hash function, i.e., by mapping features into hash keys, where multiple features can be mapped (at random) to the same hash key, and "aggregating" their counts. We compare feature hashing with the "bag of *k*-grams" approach. Our results show that feature hashing is an effective approach to reducing dimensionality on protein sequence classification tasks.

## Introduction

Many problems in computational biology, e.g., protein function prediction, subcellular localization prediction, etc., can be formulated as sequence classification tasks [1], where the amino acid sequence of a protein is used to classify the protein in functional and localization classes.

Protein sequence data contain intrinsic dependencies between their constituent elements. Given a protein sequence **x **= *x*_0_, ⋯, *x*_*n*-1 _over the amino acid alphabet, the dependencies between neighboring elements can be modeled by generating all the contiguous (potentially overlapping) sub-sequences of a certain length *k*, *x*_*i*-*k*_, ⋯, *x*_*i*-1_, *i *= *k*, ⋯, *n*, called *k*-grams, or *sequence motifs*. Because the protein sequence motifs may have variable lengths, generating the *k*-grams can be done by sliding a window of length *k *over the sequence **x**, for various values of *k*. Exploiting dependencies in the data increases the richness of the representation. However, the fixed or variable length *k*-gram representations, used for protein sequence classification, usually result in *prohibitively *high-dimensional input spaces, for large values of *k*. Applying data mining algorithms to these input spaces may be intractable due to the large number of dimensions. Hence, using dimensionality reduction techniques can be crucial for the performance and the complexity of the learning algorithms.

**Figure 1:**
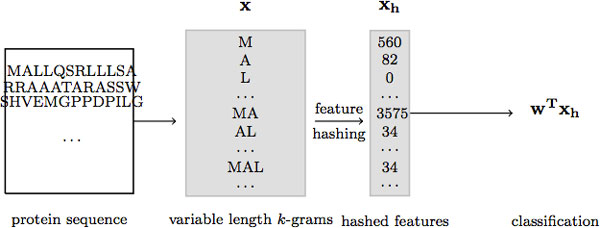
**Feature hashing on sparse high-dimensional feature spaces**. Feature hashing is performed to reduce very high dimensions to mid-size dimensions, which does not significantly distort the data.

Models such as Principal Component Analysis [2], Latent Dirichlet Allocation [3] and Probabilistic Latent Semantic Analysis [4] are widely used to perform dimensionality reduction. Unfortunately, for very high-dimensional data, with hundreds of thousands of dimensions (e.g., 160, 000 4-grams), processing data instances into feature vectors at runtime, using these models, is computationally expensive, e.g., due to *inference *at runtime in the case of LDA. A less expensive approach to dimensionality reduction is feature selection [5,6], which reduces the number of features by selecting a subset of the available features based on some chosen criteria. In particular, feature selection by average mutual information [7] selects the top features that have the highest average mutual information with the class. Recently, a new approach to dimensionality reduction, called feature hashing (or random clustering) has been introduced for text classification [8-11]. Feature hashing offers a very inexpensive, yet effective, approach to reducing the number of features provided as input to a learning algorithm, by allowing random collisions into the latent factors. Specifically, the original high-dimensional space is "reduced" by *hashing *the features into a low-dimensional space, using a hash function, i.e., by mapping features to hash keys, where multiple features can be mapped (at random) to the same hash key, and "aggregating" their counts. Figure 1 shows the application of feature hashing on sparse high-dimensional feature spaces. Although very effective for reducing the number of features from very high dimensions (e.g., 2^22^) to mid-size dimensions (e.g., 2^16^), feature hashing can result in significant loss of information, especially when hash collisions occur between highly frequent features with significantly different class distributions.

In this paper, we study the applicability of feature hashing to protein sequence classification and address the following main questions: (i) How effective is feature hashing on prohibitively high dimensional *k*-gram representations?; (ii) What is the influence of the hash size (i.e., the reduced dimension) on the performance of protein sequence classifiers that use hash features, and what is the hash size at which the performance starts degrading, due to hash collisions?; and (iii) How does the performance of feature hashing compare to that of the "bag of *k*-grams" approach? The results of our experiments on three protein subcellular localization data sets show that feature hashing is effective at reducing dimensionality on protein sequence classification tasks.

The paper is organized as follows. In Section 2, we discuss the related work. We provide background on feature hashing in Section 3. Section 4 presents experiments and results, and Section 5 concludes the paper.

## Related work

### Feature selection

Feature selection [5,7,12] is a dimensionality reduction technique, which attempts to remove redundant or irrelevant features in order to improve classification performance of learning algorithms. Feature selection methods have been widely used in Bioinformatics for tasks such as protein function prediction and gene prediction, where the features could be *k*-grams; microarray analysis; mass spectra analysis; single nucleotide polymorphisms (SNPs) analysis, among others (see [13] for a review).

### Topic models

Topic models, such as Latent Dirichlet Allocation (LDA) [3], Probabilistic Latent Semantic Analysis (PLSA) [4], and Latent Semantic Indexing (LSI) [14] are dimensionality reduction models, designed to uncover hidden *topics*, i.e., clusters of semantically related words that co-occur in text documents. LSI uses singular value decomposition to identify topics, which are then used to represent documents in a low dimensional "topic" space. LDA models each document as a mixture of topics (drawn from a conjugate Dirichlet prior), and each topic as a distribution over the words in the vocabulary. LDA has recently emerged as an important tool for modeling protein data. For example, Airoldi et al. [15] proposed the mixed membership stochastic block models to learn hidden protein interaction patterns. Pan et al. [16] used LDA to discover latent topic features, which correspond to hidden structures in the protein data, and input these features to random forest classifiers to predict protein interactions. However, topic models are computationally expensive, for example, LDA requires *inference *at runtime to estimate the topic distribution.

### Feature abstraction

Feature abstraction methods [17] are designed to reduce a model input size by grouping "similar" features into clusters of features. Specifically, feature abstraction learns an abstraction hierarchy over the set of features using hierarchical agglomerative clustering, based on the Jensen-Shannon divergence. A *cut *or level of abstraction through the resulting abstraction hierarchy specifies a compressed model, where the nodes (or abstractions) on the cut are used as "features" in a classification model. Silvescu et al. [17] used feature abstraction to simplify the data representation provided to a learner on biological sequence classification tasks.

### Feature hashing

Shi et al. [8] and Weinberger et al. [9] presented hash kernels to map the high-dimensional input spaces into low-dimensional spaces for large scale classification and large scale multitask learning (i.e., personalized spam filtering for hundreds of thousands of users), respectively. Ganchev and Dredze [18] empirically showed that hash features can produce accurate results on various NLP applications. Forman and Kirshenbaum [10] proposed a fast feature extraction approach by combining parsing and hashing for text classification and indexing. Hashing techniques have been also used in Bioinformatics. For example, Wesselink et al. [19] applied hashing to find the shortest contiguous subsequence that uniquely identifies a DNA sequence from a collection of DNA sequences. Buhler and Tompa [20] applied Locality-Sensitive Hashing (LSH) [21], a random hashing/projection technique, to discover transcriptional regulatory motifs in eukaryotes and ribosome binding sites in prokaryotes. Furthermore, Buhler [22] applied LSH to find short ungapped local alignments on a genome-wide scale. Shi et al. [8] used hashing to compare all subgraph pairs on biological graphs.

### Markov models

In the context of protein sequence classification, it is worth mentioning the fixed and variable-order Markov models (MMs), which capture dependencies between neighboring sequence elements. MMs are among the most widely used generative models of sequence data [23]. In a *k*^th ^order MM, the sequence elements satisfy the *Markov property*: each element is independent of the rest given the *k *preceding elements. One main disadvantage of MMs in practice is that the number of parameters increases exponentially with the range *k *of direct dependencies, thereby increasing the risk of *overfitting*. Begleiter et al. [24] (and papers cited therein) have examined methods for prediction using variable order MMs, including probabilistic suffix trees, which can be viewed as variants of abstraction wherein the abstractions are constrained to share suffixes.

In contrast to the approaches above, we used feature hashing, a very inexpensive approach, to reduce dimensionality on protein sequence classification tasks, and compared it with the "bag of *k*-grams" approach.

## Methods

The traditional *k*-gram approaches construct a vocabulary of size *d*, which contains all *k*-grams in a protein data set. A protein sequence is represented as a vector **x **with as many entries as the number of *k*-grams in the vocabulary. For a protein sequence, an entry *i *in **x **can record the frequency of *k*-gram *i *in the sequence, denoted by *x_i_*. Because only a small number of *k*-grams (compared to the vocabulary size) occur in a particular sequence, the representation of **x **is very sparse, i.e., only a small number of entries of **x **are non-zero. However, storing the parameter vectors in the original input space requires O(*d*) numbers, which

### Algorithm 1 Feature Hashing

**Input**: Protein sequence **x**; hash functions *h *and *ξ*, h: S→{0,⋯,b-1},ξ: S→{±1}.

**Output: **Hashed feature vector **x**^**h **^.

**x^h^**: = [0, ⋯, 0];

**for all ***k*-gram ∈ **x do**

*i *= *h *(*k*-gram) % *b*; //Places *k*-grams into hash bins, from 0 to *b*-1.

$x_{i}^{h}=x_{i}^{h}+\xi \left(k-\text{gram}\right)$; //Updates the *i*^*th *^hash feature value.

end for

**return x**^**h **^//Records values of hash features.

may become difficult given today's very large collections of protein and DNA sequence data. Feature hashing eliminates the need for such a requirement by implicitly encoding the mapping into a hash function. Next, we briefly overview feature hashing.

### Feature hashing

Feature hashing [8-11] is a dimensionality reduction technique, in which high-dimensional input vectors **x **of size *d *are *hashed *into low-dimensional feature vectors **x**^**h **^of size *b*. The procedure for hashing a protein sequence **x **is shown in Algorithm 1 and is briefly described next (see also Figure 1). Let  S denote the set of all possible strings (or *k*-grams) and *h *and *ξ *be two hash functions, such that h: S→{0,⋯,b-1} and ξ: S→{±1}, respectively. For a protein sequence **x**, each *k*-*gram *in **x **is directly mapped, using *h*, into a hash key, which represents the index of the *k*-gram in the feature vector **x^h^**, such that the hash key is a number between 0 and *b *- 1. Note that *h *can be any hash function, e.g. hashCode() of the Java String class, or murmurHash function available online at http://sites.google.com/site/murmurhash/. Each index in **x**^**h **^stores the value ("frequency counts") of the corresponding hash feature. The hash function *ξ *indicates whether to increment or decrement the hash dimension of the *k*-gram, which renders the hash feature vector **x**^**h **^to be unbiased (see [9] for more details).

**Figure 2:**
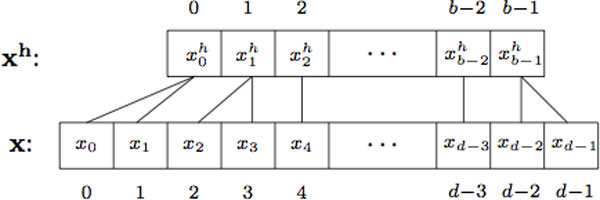
**The feature hashing representations**. The transformation of "bag of *k*-grams" into the feature hashing representations.

Thus, an entry *i *in **x**^**h **^records the "frequency counts" of *k-*grams that are hashed together into the same hash key *i*. That is,

(1) $$x_{i}^{h}=\underset{k:h\left(k\right)=i}{\sum }\xi \left(k\right)x_{k},$$

for *k *= 0, ⋯, *d *- 1 and *i *= 0, ⋯, *b *- 1. Note that in the trivial case of *ξ *≡ 1, $x_{i}^{h}$ represents the actual frequency counts (see Figure 2).

**Figure 3:**
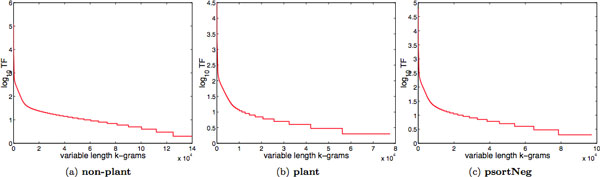
**The distribution of the variable length *k*-grams**. The variable length *k*-grams in each protein data set: (a) **non-plant**, (b) **plant**, and (c) **psortNeg**, follow a Zipf distribution, i.e., only very few *k*-grams occur with high frequency, whereas the majority of them occur very rarely.

As can be seen, multiple *k-*grams can be mapped, through *h*, into the same hash key. According to Birthday Paradox, if there are at least $\sqrt{b}$ features, then collisions are likely to happen [8], and hence, useful information for high accuracy classification could be lost through feature hashing. The *k-*grams in a collection of protein sequences typically follow a Zipf distribution, i.e., only very few *k-*grams occur with high frequency, whereas the majority of them occur very rarely (see Figure 3). Because hash collisions are independent of *k-*gram frequencies, most collisions are likely to happen between infrequent *k-*grams. Weinberger et al. [9] have proven that, for a feature vector **x **such that ||**x**||_2 _= 1, the length of **x **is preserved with high probability, for sufficiently large dimension (or hash size) **b **and sufficiently small magnitude of **x**, i.e., ||**x**||_∞ _(lower and upper bounds are theoretically derived).

However, for many practical applications, the value of *b *can be smaller than the theoretical lower bound. This may be problematic as the smaller the size of the hash vector **x**^**h **^becomes, the more collisions occur in the data. Even a single collision of very high frequency words with different class distributions, can result in significant loss of information. Next, we empirically study the applicability of feature hashing on a protein subcellular localization prediction task.

### Experiments and results

We used three protein subcellular localization data sets in our study: **psortNeg **introduced in [25] and available online at http://www.psort.org/dataset/datasetv2.html, and **plant**, and **non-plant **introduced in [26] and available online at http://www.cbs.dtu.dk/services/TargetP/datasets/datasets.php. The **psortNeg **data set is extracted from PSORTdb v.2.0 Gram-negative sequences, which contains experimentally verified localization sites. Our data set consists of all proteins that belong to exactly one of the following five classes: *cytoplasm *(278), *cytoplasmic membrane *(309), *periplasm *(276), *outer membrane *(391) and *extracellular *(190). The total number of proteins in this data set is 1444. The **plant **data set contains 940 proteins belonging to one of the following four classes: *chloroplast *(141), *mitochondrial *(368), *secretory pathway/signal peptide *(269) and other (consisting of 54 proteins with label nuclear and 108 examples with label cytosolic). The **non-plant **data set contains 2738 proteins, each in one of the following three classes: *mitochondrial *(361), *secretory pathway/signal peptide *(715) and *other *(consisting of 1224 proteins labeled nuclear and 438 proteins labeled cytosolic).

### Experimental design

Our experiments are designed to explore the following questions: (i) How effective is feature hashing on prohibitively high-dimensional *k*-gram representations?; (ii) What is the influence of the hash size on the performance of biological sequence classifiers that use hash features, and what is the hash size at which the performance starts degrading, due to hash collisions?; and (iii) How does the performance of feature hashing compare to that of the "bag of *k*-grams" approach?

To answer these questions, we proceeded with the following steps. We first preprocessed the data by generating all the *k*-grams from each collection of sequences, i.e., generating all the contiguous (potentially overlapping) sub-sequences of length *k*, for various values of *k*. This was done by sliding a window of length *k *over sequences in each data set. Note that if a *k*-gram does not appear in the data, it was not considered as a feature. The number of unique *k*-grams is exponential in *k*. However, for large values of *k*, many of the *k*-grams may not appear in the data (and, consequently, their frequency counts are zero).

Given a protein sequence **x**, we applied feature hashing in two settings as follows: (i) We first generated all the *k*-grams of a fixed length *k*, where *k *= 3. Each such *k*-gram was then *hashed *into a hash key. We refer to this setting as the fixed-length *k*-grams; (ii) We then generated all the *k*-grams of various lengths *k*, for values of *k *= 1, 2, 3, and 4. Thus, this setting uses the union of *k*-grams, for values of *k *ranging from 1 to 4. Each such *k*-gram (i.e., unigram, 2-gram, 3-gram, or 4-gram) was *hashed *into a hash key. We refer to this setting as the variable-length *k*-grams.

We trained Support Vector Machine (SVM) classifiers [27] on hash features, in both settings, fixed-length and variable-length *k*-grams, and investigated the influence of the hash size on the performance of the classifiers. Specifically, we trained SVM classifiers for values of the hash size (i.e., the reduced dimension) ranging from 2^10 ^to 2^22^, in steps of 1 for the powers of 2, and compared their performance.

Furthermore, we applied feature hashing to sparse high-dimensional variable-length *k*-gram representations to reduce the dimensionality to a mid-size *b*-dimensional space, e.g., *b *= 2^16 ^or b = 2^14^, and compared the performance of SVM classifiers trained using hash features with that of SVM classifiers trained using "bag of *k*-grams".

Specifically, the feature representations used in each case are the following:

• a bag of *d *variable-length *k*-grams (where all the variable-length *k*-grams are used as features). This experiment is denoted by *baseline*.

• a bag of *b *hash features obtained using feature hashing over all *d *variable-length *k*-grams, i.e., for each *k*-gram, feature hashing produces an index *i *such that *i *= *h*(*k*-gram) % *b*, where *h *represents a hash function. This experiment is denoted by FH.

In our experiments, we used the LibLinear implementation of SVM, available at http://www.csie.ntu.edu.tw/~cjlin/liblinear/. As for the hash function, we experimented with both the hashCode of the Java String class, and murmurHash. We found that the results were not significantly different from one another in terms of the number of hash collisions and classification accuracy. We also experimented with both ξ: S→{±1} and ξ ≡ 1 - actual counts, and found that the results were not significantly different. The results shown in the next subsection use the hashCode function and ξ ≡ 1. On all three data sets, we reported the average classification accuracy obtained in a 5-fold cross validation experiment. The results are statistically significant (*p *< 0.05). The classification accuracy is shown as a function of the number of features. The *x *axis of all figures in the next subsection shows the number of features on a log_2 _scale (i.e., number of bits in the hash-table).

## Results

### Comparison of fixed length with variable length *k*-gram representations

Table 1 shows, on the **non-plant **data set, the performance of SVMs trained using feature hashing on fixed length as well as variable length *k*-gram representations, where the hash size is set to 2^22^. As seen in the table, the performance of SVMs trained on fixed length *k*-gram representations is worse than that of SVMs trained using variable length *k*-gram representations, with *k *ranging from 1 to 4 resulting in the highest performance (the representation is denoted by (1-4)-grams). The performance of SVMs trained on fixed-length *k*-gram representations is expected to be worse than that of their counterparts trained on variable length *k*-gram representations, as protein sequence motifs have usually variable length. The performance of SVMs trained using variable length *k*-gram representations increases as we add more dependencies in the data (i.e., larger values of *k*), but starts decreasing as *k *becomes greater than 4, which may be due to *overfitting*. Similar results are obtained for the **plant **and **psortNeg **data sets (data not shown).

**Table 1:** Comparison of fixed-length with variable-length *k*-gram representations.

Bag of fixed or variable length *k*-grams	non-plant	
	
	Accuracy %	# features
1-grams	71.21	20
2-grams	70.85	400
3-grams	79.80	7999
4-grams	79.03	146598
(1-2)-grams	70.56	420
(1-3)-grams	79.69	8419
(1-4)-grams	**82.83**	**155017**
(1-5)-grams	80.09	950849

The performance of SVM classifiers trained using feature hashing on fixed length, 1-, 2-, 3-, 4-gram representations, as well as variable length, (1-2)-, (1-3)-, (1-4)-, (1-5)-grams representations, where the hash size is set to 2^22^, on the **non-plant **data set.

The number of variable length *k*-grams, for *k *ranging from 1 to 4, is 155,017. Feature hashing eliminates the need for storing the vocabularies in memory by implicitly encoding the mapping from strings to integers into a hash function. We conclude that feature hashing is very effective on prohibitively high-dimensional *k*-gram representations, which would otherwise be impractical to use. Because (1-4)-gram representation results in the highest performance, we used it for subsequent experiments.

### The influence of hash sizes on classifiers' performance and the comparison of feature hashing with baseline (i.e., the "bag of *k*-grams" approach)

**Figure 4:**
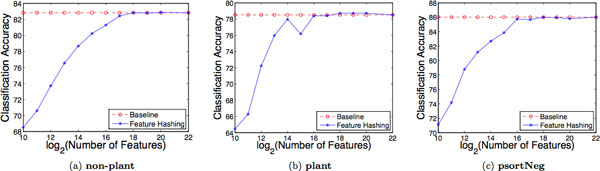
**Feature hashing vs. "bag of *k*-grams"**. Comparison of feature hashing with the "bag of variable length *k*-grams" approach, referred as baseline on the protein data sets: (a) **non-plant**, (b) **plant**, and (c) **psortNeg**, respectively, using (1-4)-grams representations.

Figures 4a, b, and 4c show the influence of the hash size *b *on the performance of the SVM classifiers, trained using variable-length *k-*grams as feature representations, on the three protein data sets used in this study, **non-plant**, **plant**, and **psortNeg**, respectively. The values of *b *range from 2^10 ^to 2^22^. The figures also show the results of the comparison of feature hashing (FH) with *baseline *on the same data sets.

As can be seen in the figures, as the hash size *b *increases from 2^10 ^to 2^22^, the performance of SVM classifiers increases as well, due to a smaller rate of hash collisions for larger values of *b*. Table 2 shows, on all three data sets used, the number of unique features and the percentage of collisions for various hash sizes. The number of unique features is calculated as the number of non-empty entries in the hash vector, and the number of collisions as the number of entries with at least one collision. Note that the percentage of collisions below 2^14 ^is 100%.

**Table 2:** The number of variable-length *k*-grams and the rate of hash collisions for various hash sizes.

Value of *b*	non-plant		plant		psortNeg	
	
	# features	Collisions %	# features	Collisions %	# features	Collisions %
2^22^	155017	0	111544	0	124389	0
2^20^	153166	1.21	110236	1.18	122894	1.22
2^19^	147223	5.29	107299	3.95	118871	4.64
2^18^	132754	16.30	99913	11.43	109535	13.22
2^17^	99764	45.04	82141	31.38	87618	35.66
2^16^	59358	78.53	53616	64.29	55555	68.85
2^15^	32474	95.80	31788	89.56	32075	92.02
2^14^	16384	100	16384	100	16384	100

The number of unique features (denoted as # features) and the rate of collisions on **non-plant**, **plant**, and **psortNeg **data sets, respectively, for variable length *k*-gram representations, where *k *varies from 1 to 4.

As the hash size increases beyond 2^16^, the performance of SVM classifiers does not change substantially, and, eventually, converges. For example, on the **non-plant **data set, with 2^16 ^hash size, SVM achieves 81.3% accuracy, whereas with 2^22 ^hash size, SVM achieves an accuracy of 82.83% (Figure 4a). On the **plant **data set, SVMs achieve 78.4% and 78.51% accuracy, with 2^16 ^and 2^22 ^hash sizes, respectively (Figure 4b). Furthermore, as the hash size increases beyond 2^16^, the percentage of hash collisions decreases until no collisions occur (Table 2). For all three data sets, with 2^22 ^hash size, there are no hash collisions. The performance of SVMs trained on hash features in the 2^22 ^dimensional space is matched by that of SVMs trained on hash features in the 2^18 ^dimensional space, suggesting that the hash collisions beyond 2^18 ^does not significantly distort the data.

Because 2^22 ^(= 4,194,304) highly exceeds the number of unique features, and the rate of hash collisions becomes zero, this can be regarded as equivalent to the classifiers trained without hashing, which require storing the vocabularies in memory, referred as *baseline *(or the "bag of *k*-grams") (Figure 4). Moreover, we considered 2^16 ^as the point where the performance starts degrading. Note that the vocabulary sizes, i.e., the number of unique variable length *k*-grams, for **non-plant**, **plant**, and **psortNeg**, are 155017, 111544, and 124389, respectively.

We conclude that, if feature hashing is used to reduce dimensionality from very large dimensions, e.g., 2^22 ^to mid-size dimensions, e.g., 2^16^, the hash collisions do not substantially hurt the classification accuracy, whereas if it is used to reduce dimensionality from mid-size dimensions to smaller dimensions, e.g., 2^10^, the hash collision significantly distort the data, and the corresponding SVMs result in poor performance. Also, feature hashing makes it possible to train SVMs that use substantially smaller number of dimensions compared to the baseline, for a small or no drop in accuracy, for example, for a hash size of 2^16 ^= 65536 (compared to 155017 variable-length *k*-grams on the **non-plant **data set).

## Conclusion

We presented an application of feature hashing to reduce dimensionality of very high-dimensional feature vectors to mid-size feature vectors on protein sequence data and compared it with the "bag of *k*-grams" approach.

The results of our experiments on three protein subcellular localization data sets show that feature hashing is an effective approach to dealing with prohibitively high-dimensional variable length *k*-gram representations. Feature hashing makes it possible to train SVM classifiers that use substantially smaller number of features compared to the approach which requires storing the vocabularies in memory, i.e., the "bag of *k*-grams" approach, while resulting in a small or no decrease in classification performance.

Because recent advances in sequencing technologies have resulted in an exponential increase in the rate at which DNA and protein sequence data are being acquired [28], the application of feature hashing on biological sequence data advances the current state of the art in terms of algorithms that can efficiently process high-dimensional data into low-dimensional feature vectors at runtime.

In the future, it would be interesting to investigate how the performance of hash kernels compares to that of histogram-based motif kernels for protein sequences, introduced by Ong and Zien [29], and the mismatch string kernels for SVM protein classification introduced by Lesli et al. [30]. Along the lines of dimensionality reduction, it would be interesting to compare the performance of feature hashing with that of feature abstraction [17] on protein sequence classification tasks. Furthermore, another direction is to apply feature hashing to other types of biological sequence data, e.g., DNA data, and other tasks, e.g., protein function prediction.

## Competing interests

The authors declare that they have no competing interests.

## Authors' contributions

All authors have read and approved the final manuscript.
